# Effect of bone morphogenetic protein-2/hydroxyapatite on ankle fusion with bone defect in a rabbit model: a pilot study

**DOI:** 10.1186/s13018-020-01891-4

**Published:** 2020-08-28

**Authors:** Le Hoang Nam Dang, Kwang Bok Lee

**Affiliations:** Department of Orthopedic Surgery, Jeonbuk National University Medical School, Research Institute of Clinical Medicine of Jeonbuk National University-Biomedical Research Institute of Jeonbuk National University Hospital, Jeonbuk National University Hospital, 634-18, Keumam-dong, Jeonju-shi, Jeonbuk South Korea

**Keywords:** Ankle fusion, Bone defect, Autogenous bone, rhBMP-2, Hydroxyapatite

## Abstract

**Background:**

Revision ankle-fusion surgery after a failure of total ankle arthroplasty has a problem with bone-defect management by implant removal. For the reconstruction of bone defects, autogenous bone often causes minor and major complications. Recombinant human-bone morphogenetic protein-2 (rhBMP-2) plays essential roles in bone regeneration strategies, and hydroxyapatite (HA) is beneficial as the rhBMP-2 carrier. In this study, we evaluate whether rhBMP-2/HA can replace autogenous bone in a rabbit ankle-fusion model with distal tibia bone defect.

**Methods:**

The bone defect was created in the distal tibia. The ankle fusion was performed by a cannulated screw from lateral malleolus and various treatments on bone defect. Thirty male white New Zealand rabbits were divided into three groups of 10 animals on each group dependent on treatment methods as control group (no treatment into defect), auto-bone group (autogenous bone treatment), and rhBMP-2/HA group (40 μL of 1 μg/mL rhBMP-2/100 μL HA). Bone formation on defect and the union of the ankle joint were evaluated by X-ray, micro-CT, and histological analysis at 8 weeks and 12 weeks, postoperatively.

**Results:**

Radiographic assessment found the control and auto-bone groups still had the bone defect present, but rhBMP-2/HA group showed complete replacement of the defect with newly formed bone at 12 weeks. Micro-CT showed significantly higher new bone formation within the defect in the rhBMP-2/HA group than in the auto-bone and control groups at 8 weeks (*p* > 0.05 and *p* < 0.01, respectively) and 12 weeks (*p* < 0.05, *p* < 0.001, respectively). Fusion rate (%) analysis of micro-CT showed a higher percentage of union in the rhBMP-2/HA group than in the auto bone and control groups at 8 weeks (*p* > 0.05, *p* < 0.001, respectively) and 12 weeks (*p* < 0.001 and *p* < 0.001, respectively). The histological showed the highest osteointegration between distal tibia and talus in the rhBMP-2/HA group at 12 weeks.

**Conclusions:**

This study indicated that rhBMP-2/HA showed much better bone fusion than did the autogenous bone graft and was effective in promoting fusion rate and improving the quality of the ankle joint fusion.

## Background

Ankle fusion in patients with bone defects presents a huge challenge to favorable reconstruction. These defects frequently occur after failed total-ankle replacement. The bone loss happens at revision arthroplasty procedures including the removal of implants and the bone preparation required before performing ankle fusion as well [1, 2].

Recently, the priority option for restoring bone defects has been autogenous bone grafting. Autograft bone to fill in bone defect sites not only provides support but also improves the biological repair of the defect. Autograft bone plays a role as osteogenic, providing osteoblasts, osteocytes, and a mineral and collagen scaffold for native cells and bone healing. Importantly, it is also osteoinductive, bringing growth factors and matrix proteins as well as signaling molecules of bone growth [3]. However, associated with autogenous harvesting, early complication rates were obtained. The minor and major complications can include persistent pain, leaving a defect at the site of extraction, blood loss, surgical site infection, hematoma, and finally the limited amount of bone in a high-risk patient [4, 5].

Bone morphogenetic proteins (BMPs), the family of osteoinductive proteins in bone matrix [6], which is as a member of transforming growth factors-β (TGF- β). It demonstrated that the strong ability to activate differentiation of mesenchymal stem cells and stem cells into osteogenic cells, which are good for producing bone. Among these proteins, recombinant human BMP-2 (rhBMP-2) possesses proliferative and chemotactic properties, acting by inducing differentiation of non-committed mesenchymal cells into osteoblast and chrondroblastic lineage [7, 8]. And it is a powerful mediator of vascular endothelial growth-factor activity. The carrier is necessary for the effectiveness of BMP-2. The carriers commonly provide an osteoconductive matrix that stabilizes the release of BMPs and thereby lowers the required dose [9]. Among these types of carriers, hydroxyapatite (HA) is an osteoconductive material. It was proved to be a strong candidate carrier for delivery of BMPs, not only because it is osteoconductive but also because it increases the delivery of growth factors [10, 11].

A lot of animal and human studies in spine and fracture surgery have shown the successful use of rhBMP-2 to induce and improve bone regeneration for different types of bone defects [12–15], but there have been few studies performed in foot ankle surgery, especially in ankle fusion with a bone defect model.

In this study, we investigated the combination of rhBMP-2 with HA as a substitute for autogenous bone in a rabbit ankle-fusion model with a distal tibia bone defect.

## Methods

### Experimental design

The selection of experimental animals, their management, and the surgical protocols were authorized by the Institutional Animal Care and Use Committee of the Jeonbuk National University Laboratory Animal Center, Jeonju, South Korea (approved number: CBNU 2017-0011). Thirty male New Zealand white rabbits (body weight around 2 kg) were used. The ankle joints were divided into three groups of 10 rabbits which had different treatments of a distal tibia defect: control group (only defect with no treatment), auto-bone group (autogenous bone treatment), and rhBMP-2/HA group (40 μL of 1 μg/mL rhBMP-2 with 100 μL HA treatment). Four ankle joints containing samples from each group were collected at 8 weeks for micro-computed tomography (micro-CT) and histological assessment. The remaining six samples from each group were assessed with plain radiographs immediately postoperation and at 4, 8, and 12 weeks postoperation, and the rabbits sacrificed at 12 weeks for micro-CT and histological assessment.

### Materials preparation

Autogenous bone was harvested from the distal femur. The lateral distal femoral metaphysis was surgically exposed by incisions through the skin and fascia lata. The periosteum was elevated by performing a full-layer incision with an #11 blade and a periosteal elevator, and the periosteum was gently dissected from the bone. Trephine bur was used to harvest autogenous bone with a diameter of 5 mm and a height of 8 mm (same volume as the volume of the defect).

The rhBMP-2 used in this study was provided by Daewoong Pharmaceutical Co., Ltd. (Novosis®-dent, Seoul, South Korea). Lyophilized 0.1 mg rhBMP-2 was dissolved in 1 ml of distilled water. The dose of rhBMP-2 in this study was established following a human undergoing spine fusion. For the human, 70 kg body weight, the dose was 1.5 mg, so dose/weight was calculated as 0.0214 mg/kg. For the rabbits of around 2 kg, the dose was 0.04 mg [16, 17].

HA (Sigma-Aldrich St. Louis, Missouri, United State) was used as the carrier material. We placed 100 μL HA into the defect, and the amount of HA was calculated approximately with the volume of the bone defect.

### Animal model

The animals were kept in separate cages following standard laboratory conditions and fed a standard diet. We anesthetized the rabbits with an intramuscular injection of a 3:1 solution of ketamine hydrochloride (Yuhan, Seoul, Korea) and xylazine (Rompun, Bayer Korea, Seoul, Korea). The anterior ankle joints were shaved and sterilized with iodine, and then local anesthesia was induced by injection of 2% lidocaine (lidocaine-HCl, Hans, Seoul, Korea).

**Fig. 1:**
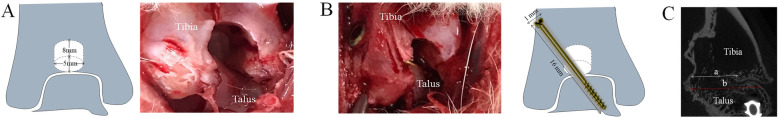
**a** Portions of the surgical procedure and **b** measurement technique for fusion rate performed on each sagittal slice through the ankle joint; **a** the short white arrow shows the length of the fused segment of joint, **b** the long red arrow shows the length of the ankle joint

The ankle joint was surgically exposed by an anterior-medial approach between the tibialis anterior and extensor hallucis longus tendon. The periosteum was then elevated by a full-layer incision with a #11 blade and a periosteal elevator, and the periosteum was gently dissected from the bone. The defects of diameter 5 mm × height 8 mm were created by a trephine bur on the center of the distal tibia from anterior to posterior (Fig. 1a). After tibia and talus cartilage was completely removed by curette, the ankle was positioned in a neutral position and ankle joints were fixed with a cannulated screw (1 × 16 mm) from the lateral malleolus to the talus-calcaneus (Fig. 1b). After the defect was irrigated by saline, the auto-bone or rhBMP-2/HA was then placed inside the defects in treatment groups, while the control group was left empty inside the defect.

The soft tissue was repositioned and then the layer was closed using 4–0 synthetic absorbable multifilament sutures (VicrylPlus Antibacterial, Ethicon, USA). Postoperative antibiotics (Amikacin, Samu Median Co., Korea; 0.15 mL/kg body weight) were provided by daily intramuscular injection for 1 week. During the postoperative stage, the animals were allowed to move freely in their cages without external support. Skin sutures were stitched out at 10 days postoperative. The rabbits in each group were sacrificed at 8 and 12 weeks postoperative.

### X-ray radiographs and micro-computed tomography analysis

Antero-posterior and lateral X-rays of the ankle joints were collected to assess new bone formation in the defect as well as consolidation status. The ankle-joint specimens including screws were harvested by trans-axial cutting 0.5 cm above the ankle-joint line and metatarsal joint dissection. Biopsy specimen b was quantitatively analyzed using a micro-CT (Skyscan 1076, Skyscan, Belgium) at 100 kV and 100 μA, with 240 ms of exposure time. The specimens were immersed in 10% neutral buffer formalin solution during the time of imaging. The bone formation volumes in the defect were estimated by the phantom in Hounsfield units (HU) using the CTAn software (Skyscan). The images obtained from micro-CT were reconstructed by the DataViewer software (Bruker, Belgium). The reconstructed images were analyzed with CTAn software. The area to be analyzed from the reconstructed images for the trabecular was specified by the region of interest (ROI), which is typically selected inside a defect border. ROI selection was achieved using a freehand drawing tool, and auto-interpolation between the different ROI levels was produced for the total volume of interest (VOI) for all frames selected.

The CT scan images of the sagittal 2-mm thick reformatted slices measured the length of the joint surface and the fused portion of the joint (Fig. 1c). The sum of the lengths of the fused segments on all slices was then divided by the sum of the lengths of the joint surfaces to calculate the fusion ratio according to the technique described by Dorsey et al. [18]. Fusion of a joint segment was defined as trabeculation or calcific density crossing the former joint space. The length of the fused segment and the length of each joint on each slice was recorded, and the fusion rate (%) was calculated using the formula: fusion rate (%) = 100 × (sum of lengths of fused segments on all slices/sum of lengths of joint surface).

### Histological analysis

The fixed ankle joint blocks were immersed in Villanueva osteochrome bone-stain solution (Polysciences, Pennsylvania, USA) and dehydrated in a graded series of ethanols and acetone 100%, and then embedded in methyl methacrylate resin (Yakuri Pure Chemical Co., Japan). The coronal sections (approx. 0.5 mm thickness) were cut, ground to a 30 to 7 μm thickness, and evaluated by optical microscopy (DM2500M-Leica, Wetzlar, Germany).

### Statistical analysis

The mean values and standard deviations for new bone formation on side defect data and ankle fusion rate (%) data were calculated for each group. An independent two-sample *t* test was used for comparisons between the groups with *p* < 0.05 considered statistically significant.

## Results

All 30 rabbits survived the surgical procedure, and there were no wound infections, local inflammation, or morbidity in any of the animals.

## Radiographic evaluation

**Fig. 2:**
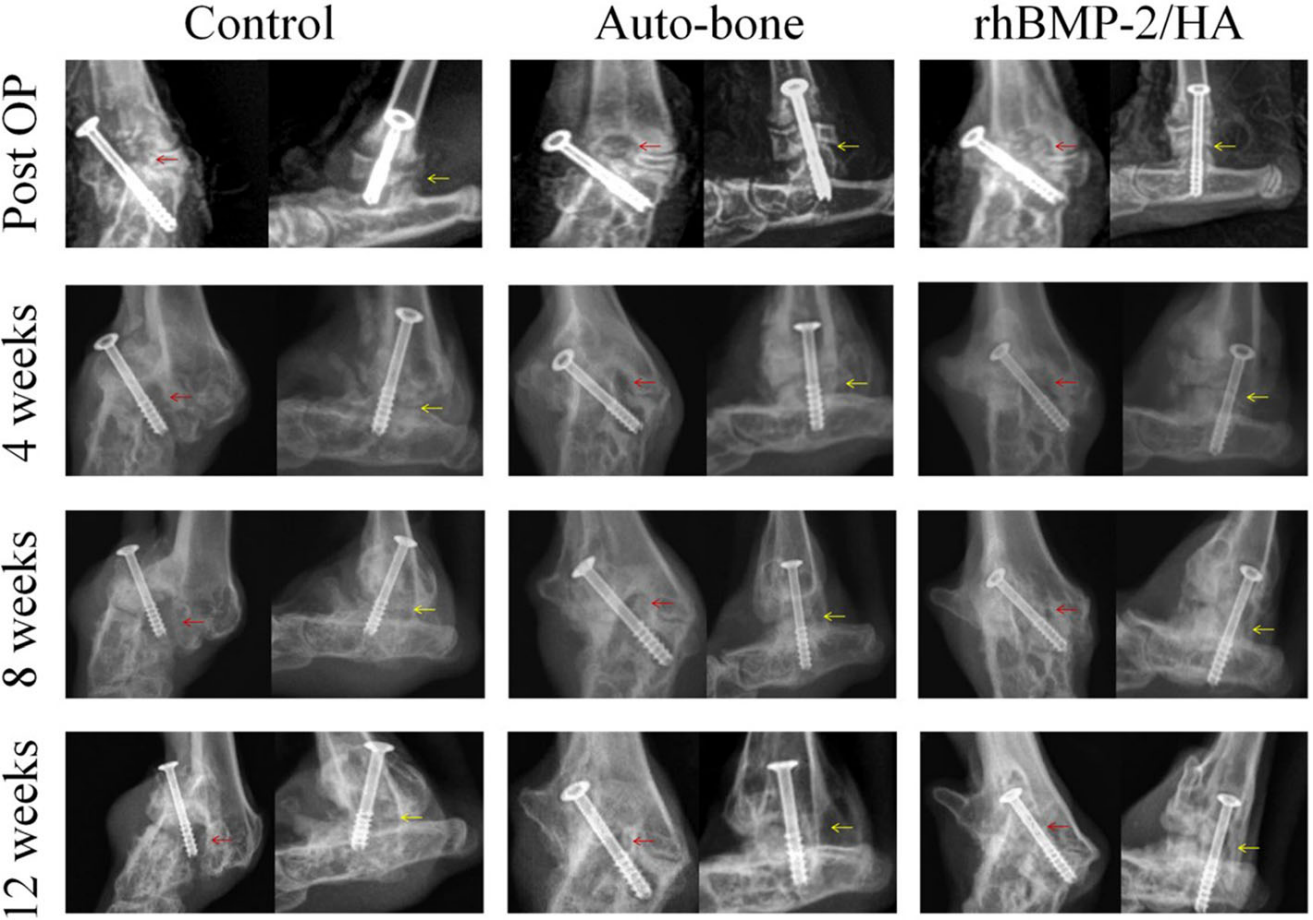
Radiographs showing the postoperative change in each group: red arrows indicate bone defect and yellow arrows indicate ankle joint space. Anteroposterior and lateral view radiographic images of the ankle joint; images were taken immediately from postoperative to 4, 8, and 12 weeks. The yellow arrows indicate the position of the ankle-joint line and the red arrows indicate the bone defect site

In all animals, new bone formation on the bone defect site was observed at 4 weeks postoperative, and the newly formed bone expansion areas at 8 weeks were larger than at 4 weeks. This difference was especially evident at 12 weeks. The bone defect in the control and auto-bone groups was still present, whereas that in the rhBMP-2/HA group was completely replaced with newly formed bone. Likewise, ankle-joint space became narrower as the time increased from 4 to 12 weeks. The consolidation in the ankle joint was solider for the rhBMP-2/HA than for the control and auto-bone groups (Fig. 2).

### Micro-CT evaluation

**Fig. 3:**
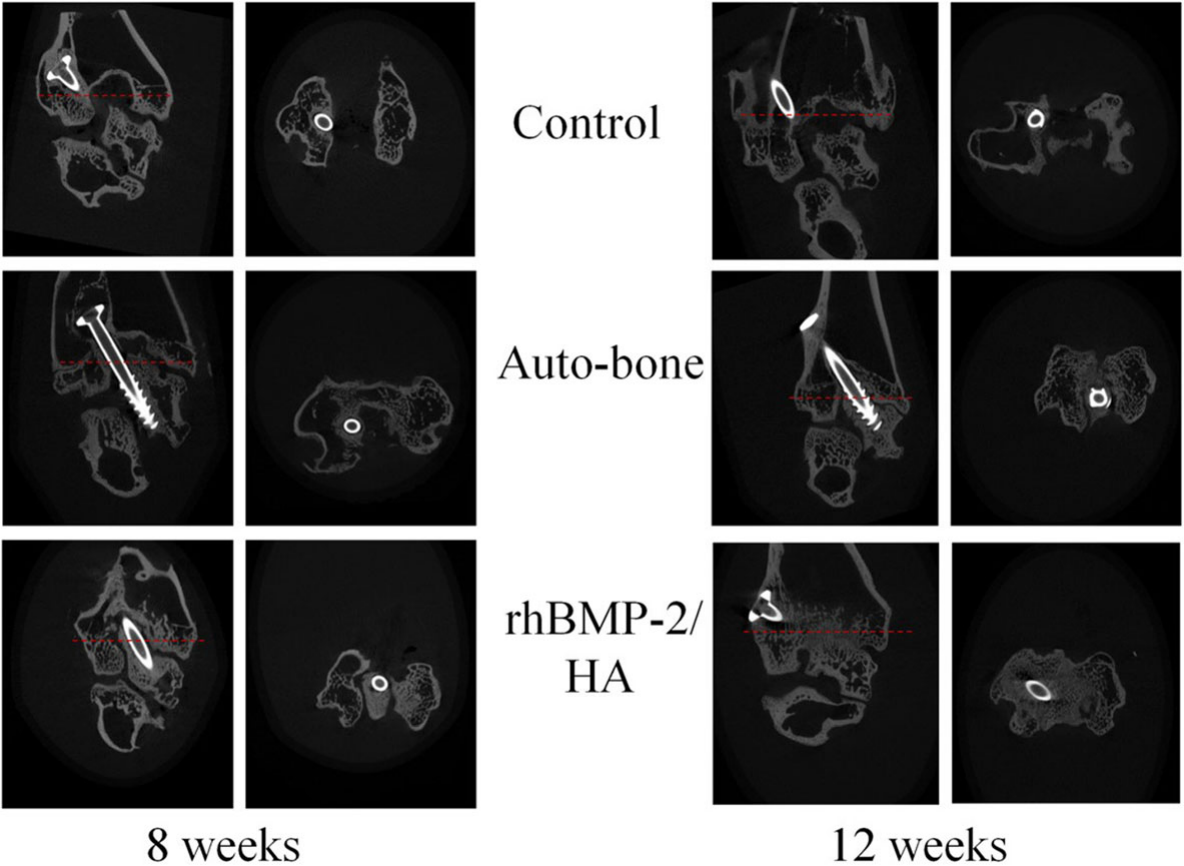
Coronal and transaxial-plane micro-computed tomography images, with red dashed lines indicating the position of the transaxial plane from the coronal plane of the ankle joint at 8 and 12 weeks postoperative

In accordance with the radiographic findings, the coronal and transaxial-plane micro-CT images with red dashed lines indicate the position of the sagittal plane from the coronal plane of the ankle joint at 8 and 12 weeks (Fig. 3). The rhBMP-2/HA group showed a higher density and volume of new bone formation on the bone defect site than did the auto-bone and control groups, and at 12 weeks postoperative, the complete union was observed in the rhBMP-2/HA group, but a partial union and nonunion were shown in the auto-bone group and control group, respectively.

**Fig. 4:**
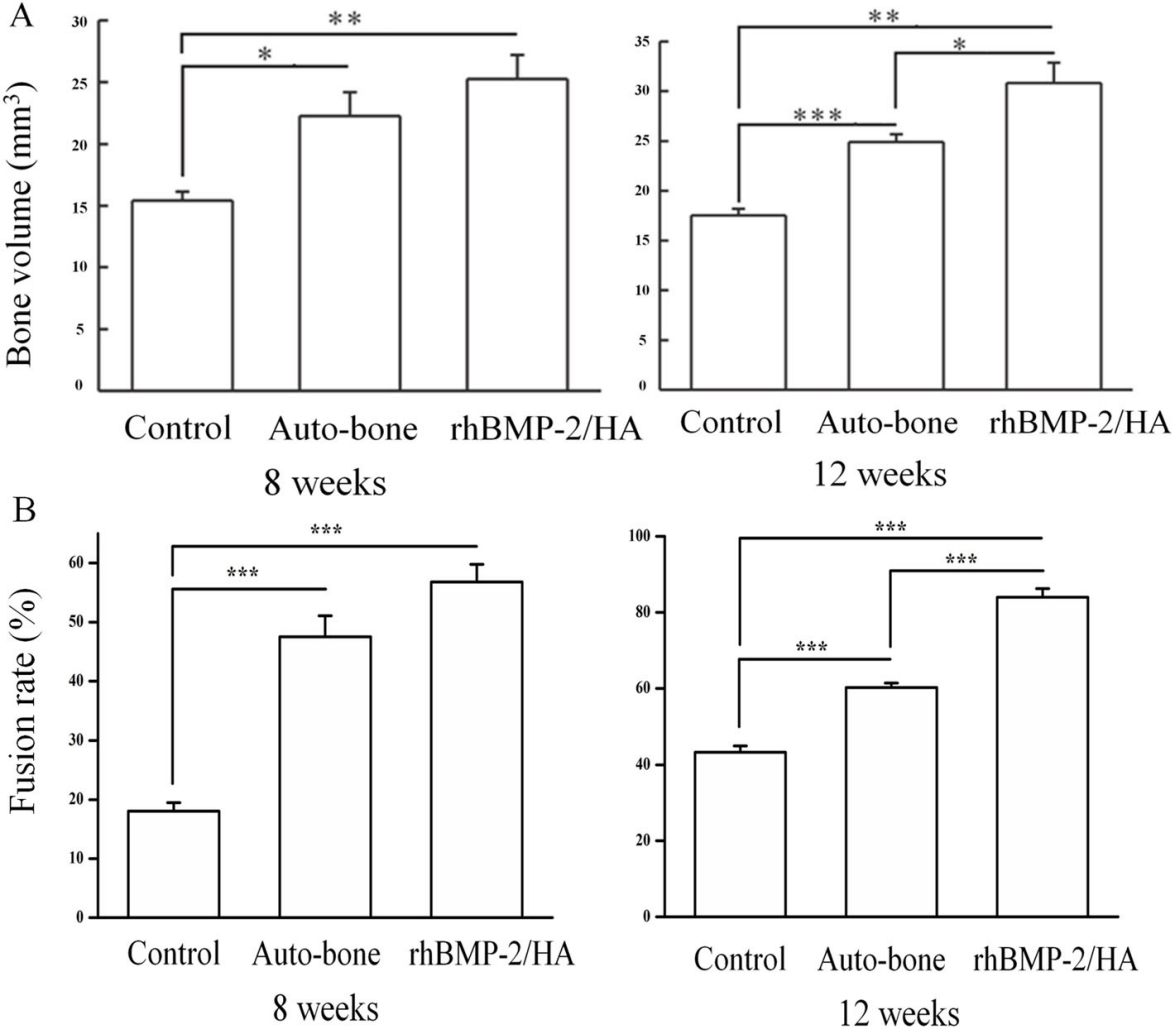
Quantitative analysis of micro-computed tomography. **a** The volume of the new bone formed on the defect at 8 and 12 weeks. **b** Parameters of the percentage fusion in the control, auto-bone, and rhBMP-2/HA groups at 8 and 12 weeks. One asterisk indicates the significant differences with *p* < 0.05, two asterisks indicate the significant differences with *p* < 0.01, and three asterisks indicate the significant differences with *p* < 0.001

At 8 weeks, the micro-CT analysis demonstrated a significantly higher volume of new bone formation in the rhBMP-2/HA and auto-bone groups than in the control group (*p* < 0.01 and *p* < 0.05, respectively). Although the volume in the rhBMP-2/HA group was higher than the auto-bone group, the difference between the two groups was not statistically significant (*p* > 0.05) (Fig. 4a).

At 12 weeks, the volume of new bone formation was significantly larger in the rhBMP-2/HA group than in the auto-bone group (*p* < 0.05), as in the results at 8 weeks. The rhBMP-2/HA and auto-bone groups showed a significantly higher volume of new bone formation than did the control group (*p* < 0.01 and *p* < 0.001, respectively) (Fig. 4a).

In accordance with fusion-rate (%) analysis by micro-CT, at 8 weeks, the ratio of bone fusion was significantly higher in the rhBMP-2/HA (58.10%) and auto-bone (48.26%) groups than in the control group (*p* < 0.001 and *p* < 0.001, respectively). A higher ratio of bone fusion was shown in the rhBMP-2/HA group than in the auto-bone group, but this difference was not statistically significant (*p* > 0.05 (Fig. 4b)). At 12 weeks, the fusion ratio was significantly higher in the rhBMP-2/HA group than in the auto-bone group (*p* < 0.001). When compared to the control group, the rhBMP-2/HA and auto-bone groups had a higher percentage of bone fusion with a significant difference (*p* < 0.001) (Fig. 4b).

### Histological evaluation

**Fig. 5:**
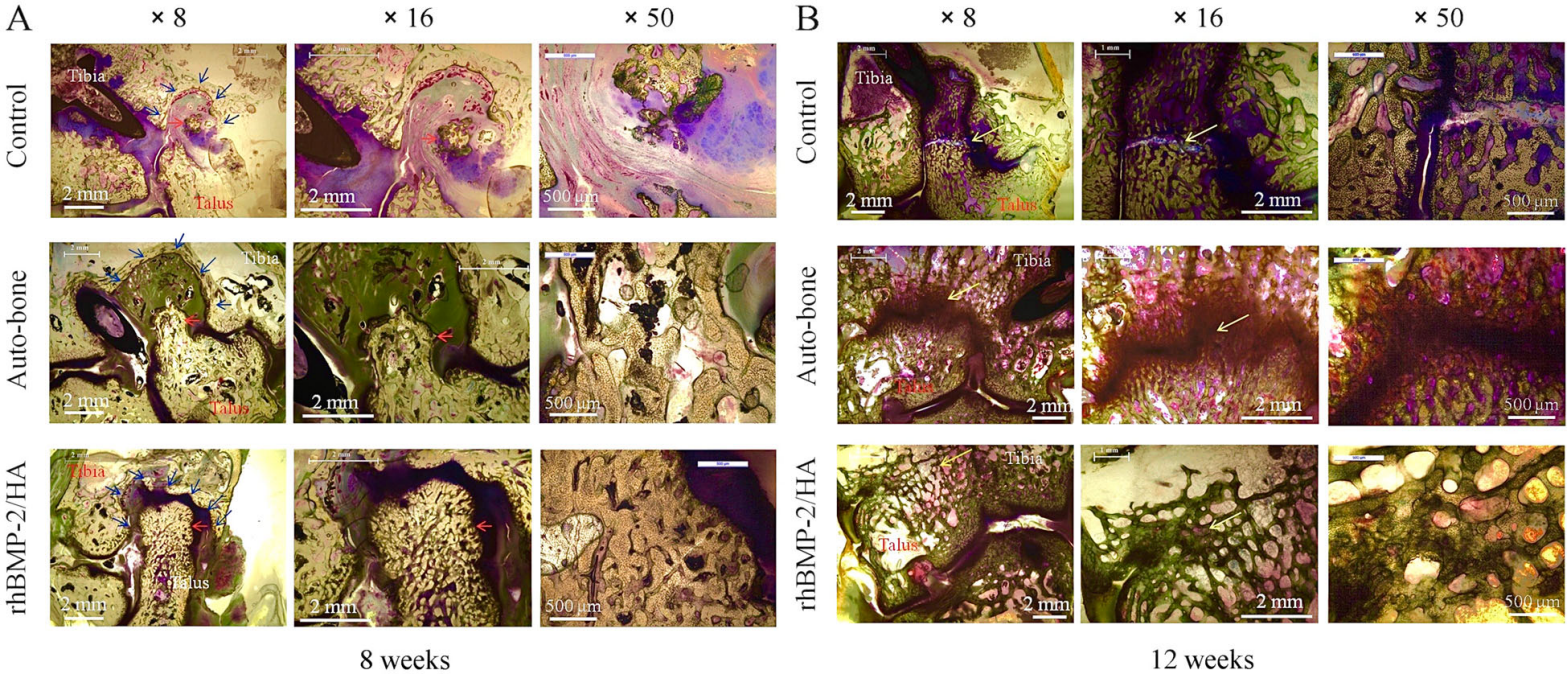
Histological images postoperative (by Villanueva bone stain). **a** At 8 weeks, red arrows indicate newly formed osteocytes on the bone defect site, and dark blue arrows indicate a margin of the bone defect. **b** At 12 weeks, yellow arrows indicate the status of consolidation between distal tibia and talus

At 8 weeks postoperative, the histological analysis showed new bone formation between distal tibia and talus in all groups. The red arrows indicate newly formed osteocytes in the bone defect site, and the dark blue arrows indicate a margin of the defect. However, the initial side of the bone defect still remained in the control group. Some new osteocytes and most of the fibrous tissue were observed on the bone defect site. The auto-bone group showed a larger osteocyte area than did the control group, but osteocyte concentration was scattered. In the rhBMP-2 group, the new bone almost completely filled the defect, and the most crowded osteocyte was shown of three groups (Fig. 5a).

At 12 weeks of postoperative, the increased new-bone formation was observed on the bone defect site in all groups, but the rhBMP-2/HA group had a different ankle-consolidation status compared with the other groups; yellow arrows indicate the consolidation status between distal tibia and talus. The control group showed nonunion of the ankle joint with the gap between the talus and distal tibia, especially in the bone defect site. A partial union appeared in the auto-bone group. Although the bone bridging connecting the talus and tibia was observed, the old sign of the gap between the talus and distal tibia remained. Especially, the rhBMP-2/HA group showed a complete union, without an old sign of a gap between the talus and distal tibia. The rhBMP-2/HA group indicated a stronger integration between talus and distal tibia than did the other groups (Fig. 5b).

## Discussion

Although the survivorship of total-ankle replacement (TAR) has improved, long-term results of TAR are not as reliable as those of total hip and knee replacements. Its overall failure rate was reported approximately 10% at 5 years, which required revision arthroplasty or conversion to a tibiotalar or tibiotalocalcaneal fusion [19]. The choice of the revision technique will mostly depend on the cause of failure, the bone defect, quality of the bone itself, and the experience and expertise of the surgeon. Based on the reviews of literature, ankle fusion is carried out more frequently than is revision replacement for failed TAR [19, 20]. It is often preferred because it is currently more reliable at providing relief from pain and gives a stable platform to bear weight even if ankle fusion limits movement and makes the limb short. The major challenge is the severe bone defect due not only to resection for exposure of the prosthesis but also to wear with secondary periprosthetic osteolysis [21]. Therefore, the development of treatment for the bone defect is a clinical challenge to foot and ankle surgeons. In spite of advances in reconstruction techniques, the complication rate has remained high. The subsequent amputation was required in 19% of patients with revision surgery [2].

Bone grafting is one of the best options to augment bone-defect regeneration in orthopedic procedures. Recently, among all clinically available grafts, autogenous bone is still being approved as the gold standard, since all important properties required in bone healing in terms of osteoconduction, osteoinduction, and osteogenesis are combined, and an autograft is completely histocompatibility with no risk of disease transmission as well [22, 23]. However, autogenous bone has largely been associated with donor site morbidity, a longer hospital stay, limited quantity, and concerns for quality in a high-risk patient [24].

In fact, the effect of rhBMP-2 on bone regeneration has been established in both preclinical studies and clinical trials with various models [25]. However, for the foot and ankle, very few studies have reported the use of rhBMP-2 and its effectiveness, especially in ankle fusion with a bone-defect model. Therefore, within this study, we combined rhBMP-2 with HA (HA plays a role as a synthetic bone substitute as well as a delivery system for rhBMP-2) and then evaluated whether rhBMP-2/HA can be an acceptable alternative to autogenous bone graft in ankle fusion with a bone defect in a rabbit model.

According to new bone formation’s promising results from radiographs, micro-CT, and histological analysis, once again, the rhBMP-2/HA delivery system shows remarkable efficiency in forming new bone on defect site when compared with other groups. These results are similar to those of many published studies that have demonstrated the effectiveness of rhBMP-2 in combination with various carrier materials in producing bone formation and healing of segmental bone defects in different types of animals and defect models. The results of BMP were equivalent to or better than those of autogenous bone-grafting [13, 14, 26]. Similar results were observed by Gerhard and co-workers following grafting of rhBMP-2 in sheep femoral defects. They showed that new bone formation in the defect sites treated with rhBMP-2 first appeared 1 month after implantation and complete bone healing at 4 months postoperative [13]. In another study, Juan Hou et al. showed that the rhBMP-2-loaded composite scaffold bridged the defects rapidly at 4 weeks, healed the defects, and presented recanalization of the bone-marrow cavity at 12 weeks [27], Elizabeth Wang et al. demonstrated the efficacy of rhBMP-2 in healing large defects in dog mandible and sheep femoral [26]. In clinical applications, in a retrospective review study by Schwartz Niles et al., the authors revealed that rhBMP-2 can heal critically large bone defects in a variety of patients, with a success rate of 84% in their study [28]. Moreover in the ankle foot surgery field, based on the results observed in a retrospective cohort study of Schuberth John et al. bone morphogenetic proteins were used in a wide variety of high-risk clinical situations in the foot and ankle and the incidence of successful bone healing was 84.21% [29]. Christophera Bibbo et al. did a total of 32 ankle fusions, which were treated with rhBMP-2 and achieved a 100% union rate in a mean time of 10 weeks [30].

Interestingly, the good results of new bone formation on a bone defect site likely relate to ankle-joint fusion results in this study. In fact, according to fusion ratio analysis by micro-CT, the percentage of bone fusion was higher in the rhBMP-2/HA group than in the auto-bone group at 12 weeks with a significant difference (*p* < 0.001), as well as at 8 weeks postoperative, although this difference was not statistically significant (Fig. 4b). Moreover, based on the classification of fusion rate on micro-CT images, Caroll Jones et al. established ankle fusion status, with nonunion as 0 to 33% fusion, partial union as 34 to 66% fusion, and complete union as 67 to 100% fusion [31]. In this study, at 8 weeks postoperative, partial union was observed for both the rhBMP-2/HA and auto-bone groups, but the control group showed nonunion. However, at 12 weeks, the rhBMP-2/HA group showed complete union, with above 84.66% fusion; in contrast, partial union was observed for the auto-bone and control groups, with 60.22% and 41.71% fusion, respectively. This result was encouraged by radiographic as well as micro-CT and histological images. In the radiographic results, the ankle-joint space became narrower with time, from 4 to 12 weeks. The consolidation status in the ankle joint was greater when comparing the control and, auto-bone groups to the rhBMP-2/HA group (Fig. 2). At 12 weeks postoperative, micro-CT images showed a complete union in the rhBMP-2/HA group, whereas the auto-bone group showed partial union, and nonunion was seen for the control group (Fig. 3). Finally, according to histological images at 12 weeks postoperative, the rhBMP-2/HA group showed complete union with stronger integration between the talus and distal tibia including new bone formation when compared with the other groups (Fig. 5b). Compared with other studies, in the results on the joint’s fusion ratio as well as union status with rhBMP-2 treatment, similar results were shown, whereas ankles receiving an auto-bone graft achieved union at a mean time of 13.3 weeks [30]. In the study of KB Lee et al., a comparison of fusion rates and time to fusion on spinal fusion, the authors demonstrated that time to fusion in the rhBMP-2 group was significantly faster than in the autograft group, and fusion rates in the rhBMP-2 group were also higher than in the autograft group, although there was no significant difference [32].

According to promising results of new bone formation as well as fusion rate (%) from radiographs, micro-CT, and histological analysis in this study, the results of the rhBMP-2/HA group were equivalent to or better than those of the auto-bone grafting group on fusion rate, quality and time to fusion as well. This difference between the two groups is most likely due to the osteoinductive properties of the graft materials as well as the osteoinduction process of the two graft materials after implantation. When autograft was implanted on a side defect, the osteoinduction process is affected by the characteristics of the autograft itself and the environment of the fusion site, such as the inflammatory response, angiogenesis, and creeping substitution. Commonly, the implanted autograft has two periods of osteoinduction. In the initial 4 weeks after implantation, osteoinduction occurs from osteocytes or osteoprogenitor cells of the implanted autograft itself, and then from the host bone after 4 weeks. Capillary invasion takes place in the new bone formed by the osteoinduction process, and then bony incorporation is completed by bone remodeling and creeping substitution. In contrast when the rhBMP-2/HA was implanted, some studies have shown a sequence of cellular events leading to the formation of new bone with all of its cellular elements. Immediately, the rhBMP-2 stimulates the stem cells to proliferate and differentiate into chondrocytes. This process takes 5 to 7 days, after which capillary invasion takes place. The chondrocytes subsequently undergo hypertrophy and become calcified, and the osteoblasts appear at the implant site. The new bone is formed at 9 to 12 days. The subsequent remodeling, formation of ossicles, and bone formation take place in the next 14 to 21 days [32–34].

The rhBMP-2 has proved to be one possible strategy for inducing bone formation, but controversy exists. The optimal therapeutic dosage, delivery system, and local circumstances for bone repairs are still under examination. Local inflammation, seroma formation, bone overgrowth, retrograde ejaculation, and increased risk of neoplastic changes are the most common complications associated with rhBMP-2 dosage [25]. The rhBMP-2 dose must be sufficient for adequate bone formation, yet not cause the known complications. Abnormal bone formation has been observed in rats after 2 weeks in critical-sized femoral bone defects treated with high doses of BMP-2 (> 150 μg/ml) [35]. Therefore, the rhBMP-2 was used at an average of 40 μg/mL based on previous research [16, 17] and the baseline dose used in human spinal fusion. No side effects such as skin infection, necrosis, or local inflammation were observed for up to 10 days; that is, the acute complications possible for wound sites did not occur. Besides, during the sample harvesting operation, we did not observe any evidence of local infection inflammations, or ectopic bone.

Much more work needs to be done if more BMPs-based tissue-engineering constructs are to become available for routine clinical use. It would require elucidation of optimal therapeutic dosage, development of more efficient carriers, and better understanding of the local bone-repair environment. In addition, there have been limited clinical trials in comparison to a large pool of preclinical studies for evaluating rhBMP-2 for routine clinical application in humans, but many promising results have demonstrated that BMPs are effective, and there is evidence that in some situations, their efficacy is comparable to or even better than autografts [36]. Our results help reinforce the above theoretical considerations, especially since this is the first investigation performed on ankle fusion with a bone defect in an animal model.

There are several limitations to this study. First, we placed a priority on imaging and histology to observe and quantify the newly formed bone on the bone defect site as well as the joint consolidation status. A mechanical strength test should be required, and this study was performed with short-term follow-up. The second limitation is related to the singular rhBMP-2 dose, carrier, and fixation method used in this study, which did not compare the effect of different rhBMP-2 doses on bone formation, such as fracture healing, bone defect, or distraction osteogenesis, and different carriers and fixation methods.

## Conclusions

This study indicated that the combination of the rhBMP-2 and HA exhibited a significantly higher quantity and quality of new bone formation on the bone defect site compared with autogenous bone graft. These results demonstrated that using rhBMP-2 with HA as carrier is a promising candidate to replace autogenous bone in treatment of ankle fusion with a bone defect. Therefore, this combination could overcome the complications of autogenous bone.

## Data Availability

The datasets supporting the conclusions of this article are included within the article.

## Abbreviations

rhBMP-2: Recombinant human-bone morphogenetic protein-2
BMP: Bone morphogenetic protein
HA: Hydroxyapatite
μCT: Micro-computed tomography
ROI: Region of interest
VOI: Volume of interest
